# Kerogen nanoscale structure and CO_2_ adsorption in shale micropores

**DOI:** 10.1038/s41598-021-83179-z

**Published:** 2021-02-16

**Authors:** Aleksandra Gonciaruk, Matthew R. Hall, Michael W. Fay, Christopher D. J. Parmenter, Christopher H. Vane, Andrei N. Khlobystov, Nino Ripepi

**Affiliations:** 1 GeoEnergy Research Centre (GERC), University of Nottingham, University Park, Nottingham, NG7 2RD UK; 2 British Geological Survey, Environmental Science Centre, Keyworth, Nottingham, NG12 5GG UK; 3 Nanoscale & Microscale Research Centre, University of Nottingham, University Park, Nottingham, NG7 2RD UK; 4 School of Chemistry, University of Nottingham, University Park, Nottingham, NG7 2RD UK; 5 Department of Mining and Minerals Engineering, Virginia Polytechnic Institute and State University, Blacksburg, VA 24060 USA

**Keywords:** Nanoscale materials, Natural gas, Scanning electron microscopy, Transmission electron microscopy, Thermodynamics, Engineering, Materials science, Energy science and technology, Carbon capture and storage, Fossil fuels

## Abstract

Gas storage and recovery processes in shales critically depend on nano-scale porosity and chemical composition, but information about the nanoscale pore geometry and connectivity of kerogen, insoluble organic shale matter, is largely unavailable. Using adsorption microcalorimetry, we show that once strong adsorption sites within nanoscale network are taken, gas adsorption even at very low pressure is governed by pore width rather than chemical composition. A combination of focused ion beam with scanning electron microscopy and transmission electron microscopy reveal the nanoscale structure of kerogen includes not only the ubiquitous amorphous phase but also highly graphitized sheets, fiber- and onion-like structures creating nanoscale voids accessible for gas sorption. Nanoscale structures bridge the current gap between molecular size and macropore scale in existing models for kerogen, thus allowing accurate prediction of gas sorption, storage and diffusion properties in shales.

## Introduction

Shale is a sedimentary rock formation containing a combination of clay, minerals and organic matter which can yield oil and/or natural gas. Many studies positively correlate gas adsorption capacity in shales with total organic content (TOC)^1–3^, also gas-in-place is generally considered to be trapped in kerogen. However, organic carbon usually constitutes only 1 to 5 wt% of shales and is surrounded by tightly packed inorganic minerals, clays, quartz and carbonates. Although intraparticle permeability restrictions may limit the rate of gas extraction, organic matter may in some cases create a continuous network over macroscopic distances for an alternative diffusion pathway. We attempt to probe the energetic distribution of adsorption sites of the whole shale material by directly measuring heat of carbon dioxide (CO_2_) adsorption in order to understand impact of inorganic and organic carbon constituents. The heat evolution with surface coverage can also provide an insight into nanoscale pore filing behavior. This is essential in understanding the gas recovery process as adsorbed volume of gas-in-place can exceed free gas volume^4^ and hydrocarbon release is greatly controlled by slow gas desorption^5^ from surface and nanopores. It is known that kerogen maturity can affect the gas sorption mechanism, ranging from absorption by dissolution into flexible immature kerogen matrices and transitioning into gas physisorption on surfaces and within the micropores of rigid mature kerogen. Classical theories such as Darcy’s^6^ and Navier Stokes^7^ fail to predict fluid flow in such small pores, while realistic molecular dynamic simulations are time consuming and still require kerogen structure of representative geometry.

Low-magnification scanning electron microscopy (SEM) images show kerogen as micron-sized isolated amorphous spots or seams^8–10^ but lack the resolution required to image nanoscale pores. Other studies have attempted to construct virtual models based on elemental and functional indicators obtained from X-ray photoelectron spectroscopy, solid state ^13^C NMR^11,12^ and Fourier transform infrared spectroscopy^13^; still the most realistic molecular models represent kerogen as large molecular weight disordered carbon structures or as an amorphous assembly of discrete molecules^14^. The large amount of published data on pore size distribution (PSD) and surface area (SA) for kerogens is based on indirect gas adsorption measurement methods, thus any conceptual model of the kerogen pore structure remains speculative in terms of pore shape, geometry and interconnectivity, also because these data have never been correlated with direct visualization of the corresponding nanoscale features. Therefore, in this study we aimed to directly explore kerogen nanostructure using focused ion beam scanning electron microscopy (FIB-SEM) and high-resolution transmission electron microscopy (TEM). Although kerogen material was previously obtained and imaged using similar technique^15^, the kerogen structure and microporosity (pore width < 2 nm^16^) remained ambiguous due to sample thickness. We isolated extremely thin specimens allowing visualization of kerogen nanoscale features.

We selected three shales, Marcellus, Barnett and Utica, that were collected from core samples of gas or oil/gas-producing wells (see “Methods”), and each differs in TOC, inorganic composition and surface parameters (Tables 1, 2). Marcellus has highest TOC, and its inorganic components are mainly silicates (illite, quartz), whereas Barnett and Utica are both low TOC shales with abundant carbonate fractions. Based on T_max_ values (Table 1) and other Rock–Eval indicators (for further details see “Methods”) Marcellus has inert post-mature kerogen and only 1% percent of TOC is pyrolysable organic carbon (PC in Table 3), Utica has oil–gas-prone mixed type II/III mature kerogen with 20% pyrolysable carbon, while Barnett has oil–gas-prone immature kerogen composed of types II and II/III moieties with 43% pyrolysable carbon. Type II are hydrogen-rich highly aliphatic hydrocarbon compounds, while type III is hydrogen-poor and oxygen-rich organics that tend to contain cyclic carbon structures^17^. Higher T_max_ value indicates greater thermal maturity as organic compounds undergo carbonification, i.e. become less aliphatic, more aromatic and lose oxygenated functional groups.

**Table 1:** Shale total organic content (TOC, wt%), thermal maturity (T_max_,°C) and mineral composition (area%).

	Barnett	Utica	Marcellus
TOC	1.65	0.28	6.70
T_max_	421	448	625
Calcite	78.07	86.06	0.58
Dolomite	1.28	2.68	0.51
Quartz	0.87	0.78	9.49
Illite	0.59	0.19	71.76
Muscovite	1.45	0.85	3.49
Apatite	2.98	1.03	0.05
Albite	0.00	0.05	1.72
Quartz–carbonate	2.39	2.24	1.06
Calcite–illite	7.96	3.77	3.89
Carbonate–clay mix	1.00	0.31	0.48
(Pyrite/chalcopyrite/sphalerite)	1.03	0.24	2.68
Other	2.38	2.81	4.30

**Table 2:** Summary of structural parameters derived from nitrogen at 196 °C and carbon dioxide at 0 °C isotherms.

	N_2_ (pore width < 200 nm)		CO_2_ (pore width 0.3–1.5 nm)	
	SA, m^2^/g	V, cm^3^/g	SA, m^2^/g	V, cm^3^/g
**Shales**				
Barnett	3.7	0.010	4.7	0.002
Utica	1.3	0.005	1.7	0.001
Marcellus	41.0	0.034	33.4	0.010
**Carbons**				
Activated carbon	963	0.447	598.8	0.185
Graphite	14.4	0.042	8.2	0.003
Mesoporous carbon	223.3	0.457	74.5	0.028
M280	40.9	0.129	9.8	0.003
**Inorganics**				
Illite	24.3	0.037	20.7	0.007
Kaolinite	7.8	0.033	5.4	0.002
Calcite	8.8	0.036	14.0	0.003
**Kerogen-enriched (KE) shales**^**a**^				
KE-Barnett (19%)	12.6	0.065	40.2	0.014
KE-Utica (2.5%)	13.8	0.047	11.2	0.004
KE-Marcellus (41%)	65.7	0.123	132.1	0.040

^a^In parenthesis, weight percent of fixed carbon measured as a mass loss at 920 °C in air flow during thermogravimetric analysis (see “Methods”).

**Table 3:** Rock–Eval data.

Shale	S1	S2	S3	PC	RC	TOC	T_max_	HI	OI
**Barnett**									
BGS	0.26	8.18	0.19	0.72	0.91	1.63	421	502	12
WL	0.24	8.22	0.24	0.70	0.96	1.66	421	495	14
**Utica**									
BGS	0.18	0.52	0.13	0.06	0.22	0.28	450	186	48
WL	0.16	0.43	0.18	0.05	0.22	0.27	445	159	67
**Marcellus**									
BGS	0.09	0.30	0.30	0.05	6.83	6.88	640	4.3	4.7
WL	0.24	0.37	0.29	0.05	6.48	6.53	609	5.7	4.4

S1—volatile hydrocarbon (HC) content, mg HC/g rock.

S2—thermally generated (cracked) hydrocarbon, mg HC/g rock.

S3—CO_2_ content, mg CO_2_/g rock.

PC—content of hydrocarbons volatilized and pyrolyzed, wt%.

RC—CO_2_ generated during oxidation representing remaining carbon, wt%.

TOC = PC + RC, wt%.

HI—hydrogen index = S2 × 100/TOC, mg HC/g TOC.

OI—oxygen index = S3 × 100/TOC, mg CO_2_/g TOC.

T_max_—temperature at which maximum of peak S2 occurs.

The differential heat (enthalpy, *Q*) of CO_2_ adsorption was analyzed using coupled volumetric-calorimetric apparatus; simultaneously measuring the volume of CO_2_ gas adsorbed and heat released. The heat of CO_2_ adsorption was measured in shales as well as different carbon materials, kaolinite and illite clays, pure calcite and kerogen-enriched (KE) shale samples. The comparison allows understanding the contribution of surface chemistry and PSD to gas adsorption. Measurements were obtained up to atmospheric pressure, thus results are relevant to the understanding of microporosity and surface heterogeneity. Further details can be found in the “Methods” section.

## Results and discussion

**Figure 1:**
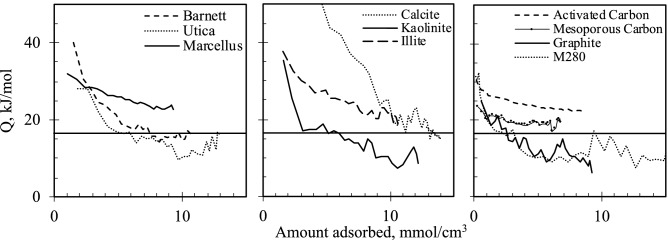
Heat of CO_2_ adsorption, horizontal line at 16.4 kJ/mol is heat of CO_2_ liquefaction.

In order to better understand the trends, differential *Q* was plotted as a function of coverage expressed as moles of adsorbed gas per micropore volume obtained using CO_2_ isotherm (Fig. 1). Heat of gas sorption is a sum of gas–solid and gas–gas interactions^18^. At low pressure, solid–gas interactions dominate, while gas–gas interactions are minimal initially increasing as multi-layer sorption filling proceeds. At saturation, gas approaches adsorptive liquid state and adsorption heat tends towards the latent heat of liquefaction (vaporization)^19^. However, at low pressure, micropores are filled and enhanced heat of adsorption can be observed due to the greater number of interactions with adjacent pore walls and gas–gas interactions that result from close-packing. Although the relative contributions from gas–gas interactions within a narrow pore will not be exactly the same as those during condensation, it serves as a guide. At low coverage Utica generated the same amount of heat as Marcellus, while Barnett produced considerably larger heat indicating these shales contain materials with strong adsorption sites. The sharp decay of the evolved heat in Utica and Barnett shales shows limited availability of these sites and greater energetic heterogeneity than in Marcellus. The fact that *Q* in Utica and Barnett approached and dropped below latent heat of CO_2_ liquefaction (16.4 kJ/mol^20^) suggests that even though full micropore volume capacity was not yet reached, adsorption occurred mainly on the flat surface, thus diluting the overall *Q* value. Indeed, both shales have very small SA and pore volume as measured by CO_2_ adsorption. The flat surface is weakly attracting and does not promote lateral gas–gas interactions, nucleation or multilayer formation at low pressures^19^, thus *Q* is only due to gas–solid interactions. This could also be the reason for *Q* stabilization at larger coverage as molecules continued adsorbing on the same surface with same energy.

The evolved heat in Marcellus decreased less steeply and was above heat of CO_2_ liquefaction. Marcellus has larger micropore volume which was shown to positively correlate with higher TOC in some shales, greater kerogen maturity^1,2,8^ and abundant fraction of illite which has accessible interlayer voids^2,21^. It is not surprising that *Q* in illite also followed similar trend. The CO_2_-illite interaction energy reported by Chen et al*.*^22^ decreases from 27 to 17 kJ/mol in 0.74 nm to 1 nm slit pores, respectively, and drops to 6.5 kJ/mol in 3 nm pore where sorption potential from adjacent pore wall is negligible, thus represents purely gas-surface interaction energy. Comparison between our measured results and all those calculated by Chen et al*.* implies that, unlike Utica and Barnett, *Q* in Marcellus and illite decreased gradually because CO_2_ was still drawn to pores up to 1 nm wide, where interactions with adjacent walls and other gas molecules tightly packed in narrow micropores maintained relatively higher *Q* values. As smaller pores are filled, progressively larger pores are gradually occupied, thus *Q* was still steadily decreasing.

**Figure 2:**
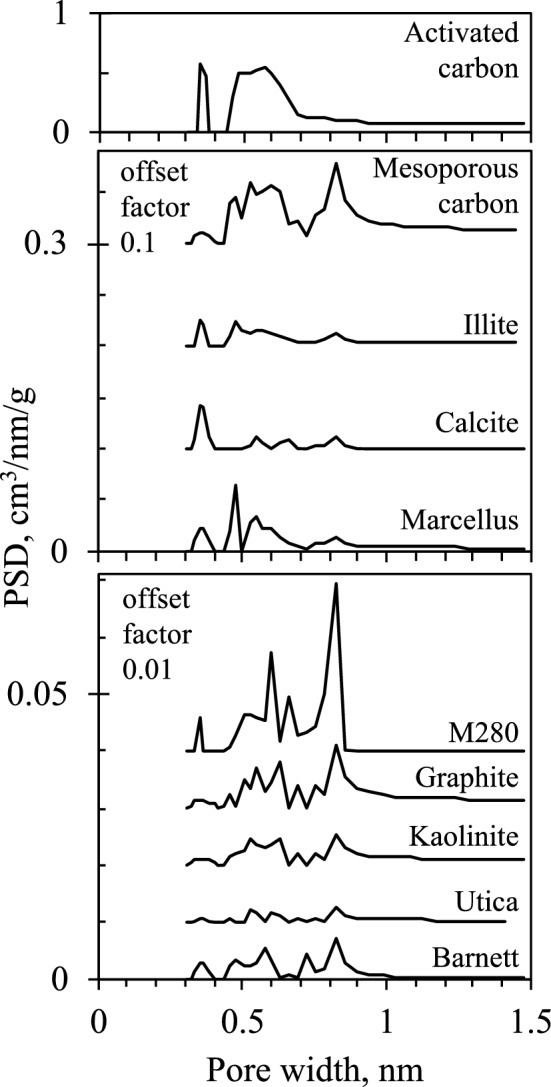
Pore size distribution.

It appears that samples having similar PSD (Fig. 2) but distinct chemical composition will adsorb gas similarly when no specific (electrostatic) gas–solid interactions are involved. For example, *Q* in kaolinite and Utica closely follow a similar trend and, like the illite-Marcellus pair, their PSD is also similar. However, Utica and Barnett are rich in calcite, yet their evolved heat is different from calcite most likely due to differences in PSD. Calcite clearly has the strongest adsorbing surface, as its *Q* is the largest among studied samples. Consequently, the initial high *Q* values in Utica and Barnett could be due to CO_2_ interactions with the calcite surface. However, a pure calcite sample also has a larger SA while its micropore volume is not that much larger than that of these two shales, which implies PSD is concentrated in the ultramicropore (< 0.7 nm) region. At lower coverages, heat of CO_2_ adsorption remained largest in Calcite as CO_2_ kept interacting with the surface and other CO_2_ molecules in narrow pores. The *Q* in Utica and Barnett declined more steeply as in-situ calcite and other substituents seem to be much tighter structures lacking porosity.

We selected carbon samples (graphite, M280 carbon black, mesoporous carbon, activated carbon) as a basic proxy for kerogen. As the real structure of kerogens is not known we chose such carbons that would have a similarly small SA and pore volume as other studied samples, and activated carbon that has large SA and micropore volume in order to assess the relative importance of chemical composition versus structural properties.

The initial *Q* values in carbon materials were lower than in inorganic or shale samples suggesting that carbon materials are weaker adsorbents. However, *Q* comparison with literature values only implies that there are no specific interactions with CO_2_ molecules. The *Q* in our carbons started at about 35 kJ/mol, corresponding to complete filling of narrowest accessible (0.4 nm) carbon infinite slit pores^23^ where both gas–gas and gas–solid interactions occur. The *Q* dropped to about 16 kJ/mol in mesoporous carbon and 12 kJ/mol in M280 and graphite, the latter being the strength of only gas-surface interactions^23^. As initial *Q* is twice that of a single surface, this suggests that CO_2_ adsorbs first in the narrow gap between two pore walls and there is no *Q* enhancement due to specific interactions with carbon surface and/or presence of closed-end pores that would increase the number of interactions with the surface.

However, at higher coverages shales, inorganic matter and carbons can adsorb CO_2_ equally as strongly since heats of adsorption approached similar values. The heats of adsorption presented in Fig. 1 show that both inorganic and organic samples can adsorb CO_2_ at similar heat and at higher coverage. For example, the curve of Marcellus shale and activated carbon (group 1) merge at about 25 kJ/mol. Utica, kaolinite, graphite and carbon black (M280) (group 2) also release similar heat in the range of 11–14 kJ/mol. Of course, group 1 adsorbs CO_2_ stronger at 9 mmol/cm^3^ coverage than group 2, but the difference can be explained by the greater presence of micropores in group 1 materials. Thus CO_2_ is exposed to a larger force field from the surrounding pore walls and adjacent CO_2_ molecules. This means that non-specific dispersion (van der Waals) forces are likely to dominate intermolecular or interatom bonding. Maia et al*.* ^23^ made the same observation for activated carbons with varying concentration of oxygen functional groups that would have specific interactions with CO_2_. It was clear that both microporosity and chemical composition played an equal role: a sample with fewer oxygen groups and larger micropore volume had similar *Q* values to a sample with lower micropore volume but more oxygen groups. These observations imply that non-specific interactions are similar in both carbon-based and inorganic-based materials. However, inorganics additionally provide adsorption sites where functional groups or cations interact with CO_2_ specifically. Consequently, in combination with confined space effect inorganics adsorb CO_2_ stronger at low coverages than carbon-based samples. However, released heat becomes similar to that of other samples at higher coverages even in calcite’s case.

**Figure 3:**
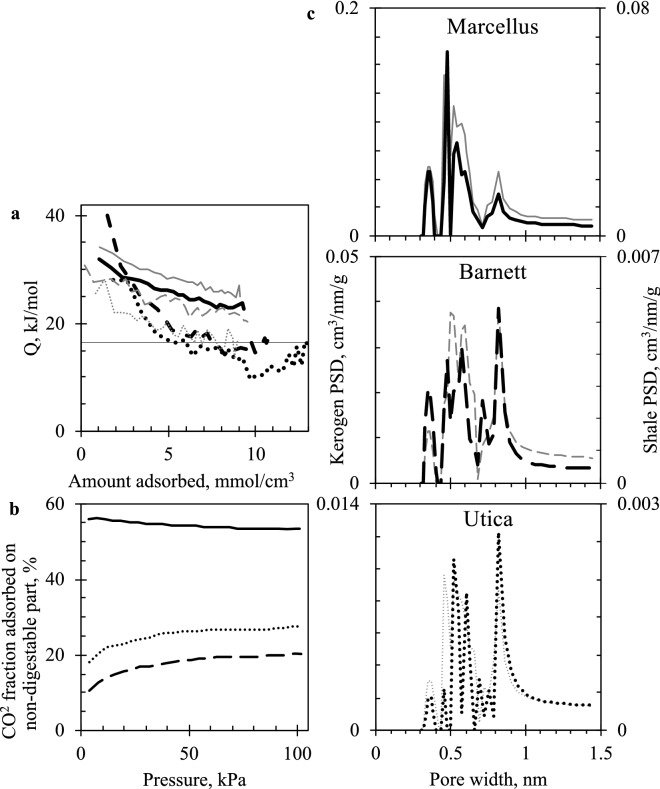
Heat of adsorption (**a**), fraction of CO_2_ adsorbed on non-digestible part of shale (**b**) and micropore size distribution comparison between shales and kerogen-enriched shales (**c**) (black thick: — Marcellus, – – Barnett, ••• Utica, grey thin: kerogen-enriched shales).

Structural properties and adsorption thermodynamics of demineralized or kerogen-enriched (KE) shales (see “Methods” for preparation procedure) suggest that significant microporosity could be concentrated within the organic part of shales (Fig. 3). Micropore volume was increased in all shales after demineralisation, however PSD remained similar. Adsorption heat of KE shales decreased with the same slope as that of untreated shales. This suggests that the micropore network was retained after treatment, and that its surface was fully accessible via the inorganic pore network in the whole shale. However, values were consistently higher by about 3 kJ/mol in Marcellus, and 5 kJ/mol in both Barnett and Utica residues, which means that the surface became more favorable as weaker adsorbing minerals were removed. The initial *Q* in KE Utica and Barnett started at lower values suggesting that the removed minerals had very strong adsorption sites; most likely calcite (and other carbonates) prior to removal during acid treatment.

By separating shale constituents into organic and inorganic components, and by knowing CO_2_ uptake in the original and demineralized shales, it is possible to calculate the fraction of CO_2_ adsorbed on kerogen-enriched (organic) and acid-digested (mineral/inorganic) parts, and compare their adsorption strength in absolute terms (Fig. 3b). Overall, acid-digestible inorganics adsorb the most CO_2_ in Barnett and Utica shales (about 70%). However, these inorganics constitute an even greater percent (93–96 wt%, see Table 4 in “Methods”) of shale sample mass. Therefore, KE residue is a slightly stronger adsorbent, which in turn suggests that a substantial fraction of the microporosity is concentrated within it. A similar situation is evident with Marcellus shale, where KE residue adsorbs about half of total CO_2_ uptake even though it constitutes only about 14 wt% of shale.

**Figure 4:**
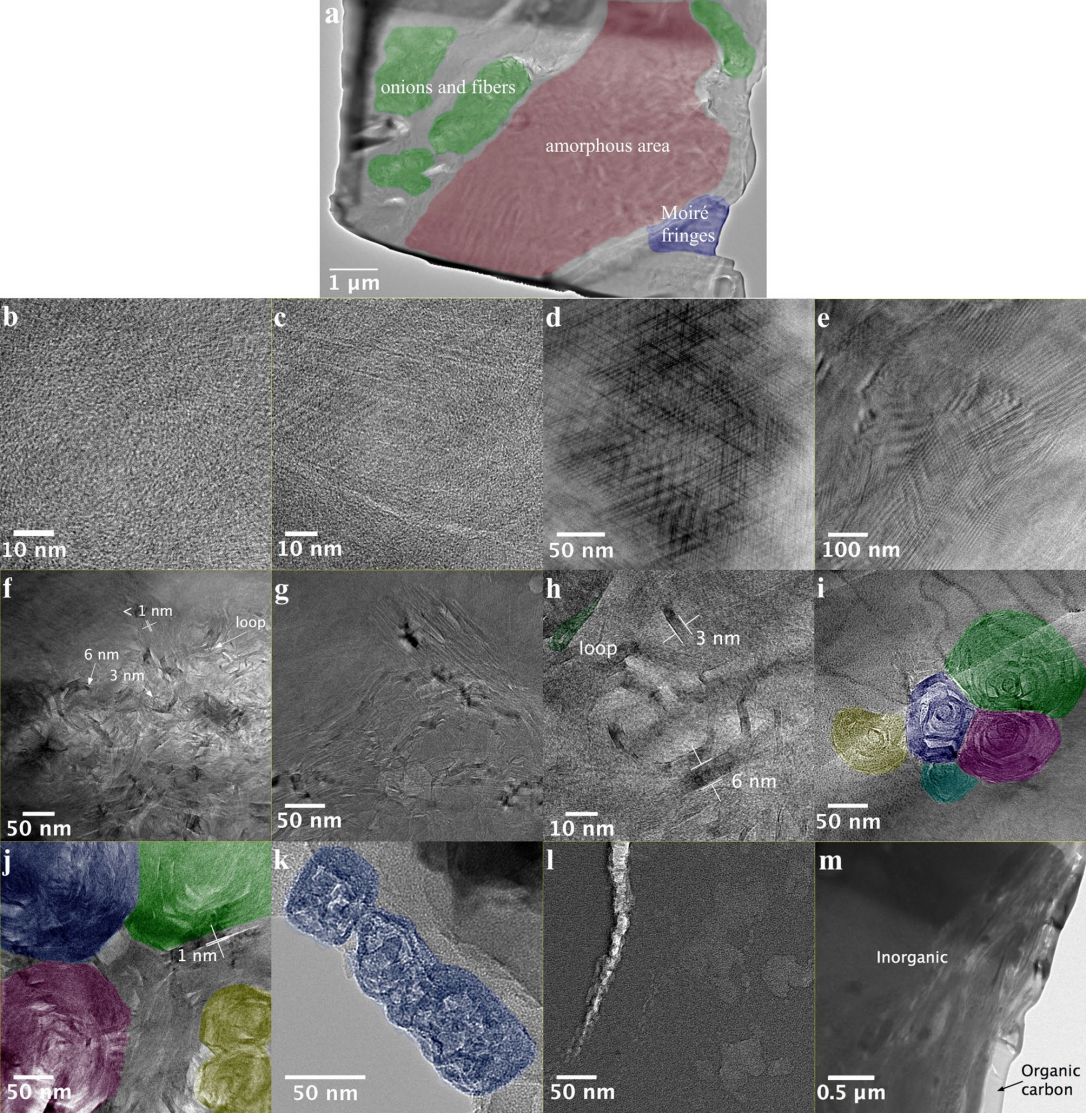
TEM images of (**a**) spatial variability of Marcellus shale lift-out; respectively, Marcellus’ and Barnett’s (**b**,**c**) amorphous structure, (**d**,**e**) Moiré fringes, (**f**,**g**) fibers, (**h**) zoomed-in region of Marcellus’s micrograph in (**f**), (**i**,**j**) onions, (**k**) kerogen-enriched Marcellus shale bulk particle, (**l**) amorphous structure of Utica’s kerogen and (**m**) Utica’s lift-out. Coloring is for the guidance to the eye.

Although the above results suggested that adsorption on a carbon surface is not as strong due to lack of specific interactions, the elevated adsorption capacity of KE shales could be due to a favorable kerogen pore structure distinct from some of the carbon samples and similar to activated carbon. Brightfield TEM images revealed that organic matter separated from Marcellus and Barnett has a structural variability within a 4 × 7 µm area (Fig. 4a) which would most likely correspond to the size of an individual organic maceral^10^. Such spatial irregularity within one maceral could be due to different catalytic and thermal effects caused by different surrounding minerals. Alternatively, the spatial irregularity could be attributed to the presence of more than one type of organic maceral that are compressed into one area. Nevertheless, images show that the organic matter consists of not only amorphous phase (Fig. 4b,c) but also a few distinct features suggesting crystalline organization with abundant void volume. Barnett’s immature kerogen has similar structures to over-mature Marcellus’ kerogen, even though microscale porosity was previously shown to be less apparent in low maturity shales^8^.

Different structures revealed by TEM imaging are summarized in Fig. 4. Moiré fringe patterns that are likely to be created by overlapping misaligned large crystalline graphitic sheets are shown in Fig. 4d,e. The 3 to 7 nm wide graphitic stacks of several layers that form long fibers can be clearly seen in Fig. 4f,g. These fibers can be either randomly intertwined, some seem flexible and folded on itself forming loops (Fig. 4h), or create high aspect ratio secondary stacks with long ~ 1 nm interspacing seen as lighter, i.e. less dense, areas. This coincides with PSD peaks at about 0.9 nm visible in CO_2_ PSDs. Similar structures can be observed in turbostratic graphite fibers, where few-layer graphite stacks misalign and split due to random rotation or tilting and folding yielding voids between fibres^24–26^. Such structures can be obtained by high temperature (~ 3000 °C) carbonization in an inert environment and higher temperature tends to create larger stacks and voids between them^27^. The stack size varies from 3 to 6 nm in both Marcellus and Barnett shales, similar to fibers obtained at higher temperatures. Similar structures made mostly of single curled layers can also be observed in activated carbons. Harris et al*.*^28^ suggested that pentagonal carbon rings present in original carbon and preserved even after 2000 °C treatment result in a curved structure that resists graphitization, thus fullerene-like models explain microporosity of such materials better than traditional slit-shaped carbon pores. This could also be the reason why even post-mature Marcellus kerogen is not graphitized and displays both amorphous and curved fiber-like phases.

The fibers also form circular structures. Loops visible in Fig. 4h possibly serve as nucleation points for secondary folds that eventually form either scrolls or carbon “onions” (Fig. 4i,j). In the first case, the whole pore volume in-between fibers should be continuous and accessible for gas, whereas in the latter case it could be completely enclosed by layers of graphitic sheets. The presence of such structures can indicate exposure to high temperatures, as similar concentric shells can be observed in graphitized carbon blacks^29^ and nanoparticles found in natural gas/air flame^30^. Similar to the kerogen onions, carbon black shells are also about 5–7 nm thick and form secondary shells when exposed to high temperatures. Within bulk KE-Marcellus particles, we also observed many similar-sized circular structures with 3–5 nm wide graphitic shells (Fig. 4k).

Brightfield TEM images showed that Utica’s carbon is completely amorphous (Fig. 4l) which is consistent with current literature descriptions stating that Utica’s organic material is dominated by amorphinite^31^. However, the heat in KE-Utica evolved more gradually than in untreated shale, indicating gas adsorption in smaller pores. The micrograph hints that there are some less dense regions and cracks (lighter grey to white), however their size is in a meso- to macro-scale region. The amorphous phase can also have micro-sized pores, however it is difficult to visualize even using high resolution TEM, when the thickness of even the thin FIB prepared layer (few 10 s of nm) is still significantly thicker than the size of the features analyzed (less than 2 nm), resulting in a 2D projection of many overlapping features in the TEM micrograph. Thus, only more defined structures such as seen in Marcellus and Barnett case would show the direct evidence of microporosity. Another limitation of the TEM technique is that information may not be statistically significant unless much larger areas or many particles are sampled. Therefore, it is possible that other phases in Utica’s kerogen are simply rare. On the other hand, microporosity might be originating in inorganic material that still makes a large fraction of the Utica shale after acid treatment. Indeed, micrographs also show voids in inorganic regions (Fig. 4m), adsorption on which could provide consistent heat release.

## Conclusions

The coupled sorption-calorimetry study indicate that strength of CO_2_ adsorption depends on both surface chemistry (gas–solid interaction) and pores size (gas–solid and gas–gas interactions). The magnitude of measured *Q* provides an insight into adsorbents strength, while the trend depends on available micropore volume and PSD. Our results suggest that adsorption occurs simultaneously on a flat surface or larger pores even though full micropore volume capacity is not reached. Inorganic materials and shales showed enhanced initial *Q* values because of strong adsorption sites that interact with CO_2_ specifically and confined pore volume where gas molecules interact with adjacent walls. In the case of carbons, the larger initial adsorption heat was only due to gas adsorbing in narrow micropores. Once the smallest pores and strongest adsorption sites were occupied all samples adsorbed CO_2_ with the similar strength regardless of their chemical composition, thus it is reasonable to assume that gas would populate the whole shale pore network. Samples that released more heat which decreased less steeply had greater pore volume which is concentrated in micropore region. This volume was likely readily available for adsorption, increasing number of gas-surface interactions and additionally promoting gas–gas interactions.

The comparison of evolved heat slope suggested the whole pore network was accessible in the original shale sample even prior to acid digestions. Although we analyzed crushed samples, which exposed more volume and surface than what would be accessible in intact and tight core, it still indicates that pores were not shielded by removed inorganic fractions.

Kerogen potentially holds a significant fraction of micropores within shales. The *Q* in KE samples was larger and decreased less steeply than that of their original shale which confirms they have larger pore volumes and are more chemically homogeneous. TEM images show that kerogens have several intricate crystalline carbon structures: amorphous phase, large scale graphitic plates, fibrous and curved onion-like structures that make nanoscale pores between graphitic stacks. The latter structures can potentially have a continuous network of pores that is accessible to fluids from outside and allows shale gas to escape. Although TEM imaging revealed distinct features of kerogen nanostructure, further screening analysis is needed to estimate prevalence of each feature quantitatively. Nevertheless, the observations significantly enrich limited library of kerogen models with complex carbon nanostructures. These morphologies are likely to govern not only gas adsorption properties, but also gas transport and mechanical behavior, thus will have implications in reservoir engineering of gas shales and development of accurate fluid flow theories.

## Methods

### Materials

All shale samples were donated by associate professor Nino Ripepi from Virginia Tech, Virginia Center for Coal & Energy Research. We selected three shales, Marcellus, Barnett and Utica, that are significant natural gas and oil producers in the United States^32^. The Mississippian-aged Barnett shale is located in the Fort Worth Basin in Texas and its thickness ranges between 91 m to greater than 300 m^33^. The Devonian-aged Marcellus and Ordovician-aged Utica shales are located in the Appalachian basin with the Marcellus shale ranging from 20 to 100 m thick^34^ while the Utica shale, which lies 1.2–1.8 km deeper than the Marcellus, is significantly thicker, up to 150 m. The Barnett and Marcellus shales are similar to each other lithologically as well as having similar mineral composition and have both been successfully and economically produced through horizontal drilling coupled with hydraulic fracturing techniques because of the brittleness of the shales^32^. The Barnett shale produces both oil as well as dry gas while the Marcellus is primarily a dry gas play. The Utica shale is considered the major source rock in the Appalachian basin^35^ and produces both oil and dry gas.

Shale cores were ground using ring and puck pulverizer into powder form and sieved through 106 µm mesh, sub-samples were taken for microcalorimetry, microscopy and sample characterisation work from the exact same batch. Graphite, mesoporous carbon (Supelco), kaolinite and calcite were purchased from Sigma Aldrich, activated carbon from Brownell, illite (illite-rich Cambrian shale from Silver Hill, Montana) from The Clay Minerals Society, M280 carbon black was donated by Cabot.

### Volumetric adsorption

Specific SA and PSD were derived from nitrogen and carbon dioxide adsorption isotherms at − 196 °C and 0 °C, respectively. Isotherms were measured on Micromeritics 3Flex surface characterization analyzer. Samples were degassed for 17 h at 110 °C in vacuum before analysis. Specific surface area was calculated using BET method, pore volume and PSD were estimated using available DFT methods stored in ASiQwin software (Quantachrome). All isotherms were analyzed using QSDFT method for N_2_ adsorption at − 196 °C in carbon slit/cylinder pores and NLDFT method for CO_2_ adsorption at 0 °C in carbon slit pores. Even if fitting errors are small, we acknowledge that carbon model may not be accurate enough representation of non-carbon materials and even organic fractions of shales due to structural and chemical complexity distinct from pure carbon surface, therefore there might be some uncertainties especially in PSD, thus we avoid overinterpretation of PSD peaks and use it for general comparison between samples.

### Adsorption microcalorimetry

**Figure 5:**
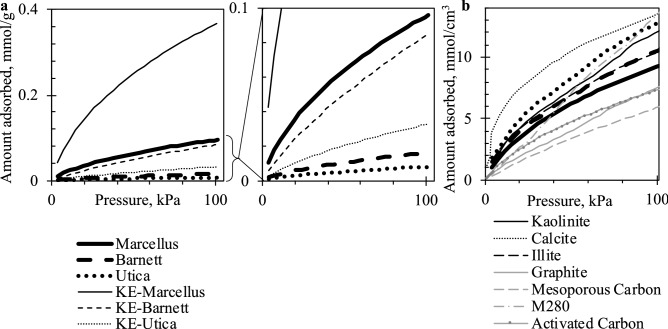
Amount of CO_2_ adsorbed in mmol/g (**a**) and in mmol/cm^3^ (**b**) at 26 °C. The data was used to plot heat of adsorption versus coverage and calculate fraction of CO_2_ adsorbed on kerogen-enriched and acid-digested parts of shales.

Heat of sorption was measured using coupled volumetric-calorimetric apparatuses. The apparatus consists of commercially available Setaram SensysEvo differential scanning calorimeter (DSC), in-house designed sample cell and Quantachrome Autosorb-iQ2 volumetric dosing system. The calorimetric transducer was detached from the main body in order to place it as close as possible to iQ sample port thus minimizing dead cell volume. The calorimeter uses heat flow rate technique and is designed using Calvet principle, i.e. sample and reference are surrounded by a cylindrical sensor that detect difference in heat flow rate to the sample and reference while both are kept at the same temperature. The DSC was calibrated by Joule effect and presented results are an average of three experiments. DSC sensitivity is 8.35 µV/mW. The 6 mm glass cell is filled with up to 2 cm of sample so that sample is within DSC measuring zone. Both sample and empty reference cells are inserted into DSC and connected to iQ through stainless steel T-adaptor via Ultra-Torr fittings. Before analysis, the sample is degassed in-situ at 110 °C for 15 h by simultaneous evacuation performed by iQ and heating by DSC. Discrete volume of gas is dosed into cells and once equilibration parameters are satisfied system advances to the next dose until maximum, atmospheric, pressure is reached. DSC records sample temperature and heat flow signal throughout the analysis. Isotherms were measured at 26 °C (Fig. 5), close to iQ’s internal temperature (25 °C) to minimize any additional calorimetric effects possibly observable due to asymmetry of sample and reference cells. Amount of heat released or absorbed due to adsorption was calculated by integrating heat flow peak. Differential *Q* (kJ/mol) was calculated by dividing this quantity with amount adsorbed at a particular dose measured by iQ. The analysis was repeated three times for each sample and data is reported as an average. Average accuracy of *Q* points is ± 1, ± 3 and ± 5 kJ/mol for Marcellus, Barnett and Utica shales, respectively.

### Kerogen enrichment

Shale samples were sonicated in 37% hydrochloric acid (HCl) solution at 60 °C for about 7 h, then left still overnight at room temperature. The HCl-digested samples were washed with distilled water, centrifuged and decanted until litmus paper showed neutral solution. The residue was stirred at 60 °C in 2:1 solution of 40% hydrofluoric (HF) and 37% HCl acids for 4 h. After the treatment samples were continuously washed with distilled water, centrifuged and decanted until solution became neutral. The fixed carbon was measured as a weight loss during oxidation at 920 °C after gas switch from N_2_ to air flow using thermogravimetric analysis. The percentages of mass loss after each treatment are shown in Table 4. After digestion with HF acid, Utica shale lost most of its fixed carbon due to mechanical loses, thus the sample treated only with HCl acid was used as kerogen-enriched sample.

**Table 4:** Percentage mass loss and amount of fixed carbon after treatments with HCl and HF acids.

	Barnett	Utica	Marcellus
Mass loss post-HCl, wt%	89	93	8
Mass loss post-HF, wt%	63	34	84
Total, %	96	95	85
Fixed carbon post-HCl, wt%	6.6	2.5	6.9
Fixed carbon post-HF, wt%	19	0.4	41

### Thermogravimetric analysis

TGA was performed on TA Instruments Q500 thermogravimetric analyzer under nitrogen atmosphere, temperature was increase to 105 °C with 10 °C/min ramping rate and kept for 15 min, the second increase was up to 920 °C with 10 °C/min ramping rate, temperature was again kept for 15 min, then flow was switched from nitrogen to air keeping temperature for another 15 min.

### X-ray diffraction (XRD)

**Figure 6:**
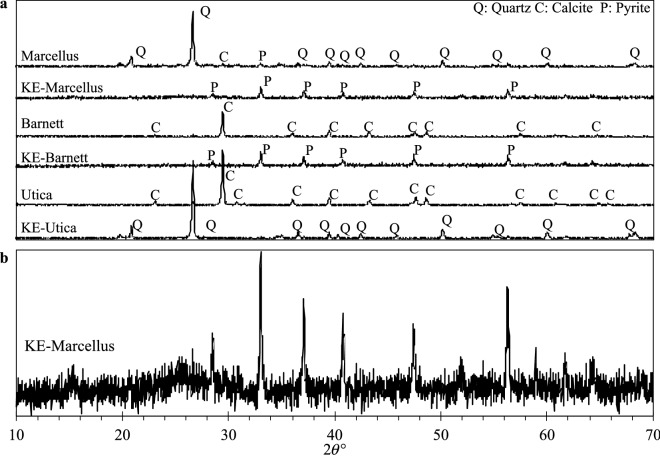
XRD patterns of shales and kerogen-enriched shales (**a**) and KE-Marcellus (**b**).

Shale composition was examined before and after acid treatment (Fig. 6) using Bruker X-ray diffractometer with Cu Kα (λ = 0.154 nm) at 40 kV and 35 mA. The 2θ range of 10°–70° was recorded with 0.02° steps. Calcite was removed from all shales and quartz was removed from Marcellus and Barnett shales leaving crystalline structure of predominantly pyrite. Utica shale was treated only with HCl acid, so its XRD pattern exposed remaining quartz. Characteristic graphite (002) peak was not expected due to insufficient carbon fraction and scarce carbon structures newly discovered in this work, meaning most carbon is non-crystalline. However, there is a slight hint of a carbon crystallinity in KE-Marcellus (Fig. 5b), which had the largest carbon fraction based on TGA data.

### Rock–Eval pyrolysis

Rock–Eval pyrolysis was performed in Weatherford Laboratories (WL) and in British Geological Survey (BGS) (Table 3). Pyrolysis was performed in standard mode (pyrolysis and oxidation as a serial process)^36^. Powdered rock samples (60 mg/dry wt) were heated isothermally at 300 °C (hold for 3 min) and then heated from 300 to 650 °C (hold for 3 min) at 25 °C/min in N_2_ atmosphere. Recovered sample was oxidized in CO_2_ free air by reheating from 300 to 850 °C at 20 °C/min. Hydrocarbons released during pyrolysis were detected using a flame ionization detector and CO and CO_2_ released during pyrolysis and oxidation detected using an infrared cell. The areas under detected peaks were used to calculate indexes shown in Table 3. Although exact value of T_max_ for Marcellus shale is reported as unreliable, the very low and bimodal S2 peak despite relatively high TOC indicates over-mature nature^37^. Additionally, Marcellus shale was indeed extracted from deep dry gas producing well. Therefore, the high T_max_ value is still expected and used quantitatively to compare with maturity of Utica and Barnett organic matter.

### Mineral liberation analysis

Mineral composition (Table 1) was identified and quantified by analyzing imaged area of polished resin imbedded particles using scanning electron microscopy (FEI Quanta 600 FEG SEM) and Mineral Liberation Analysis software by JKTech/FEI. Percent composition is taken as area fraction.

### Kerogen lift-out preparation and microscopic imaging

**Figure 7:**
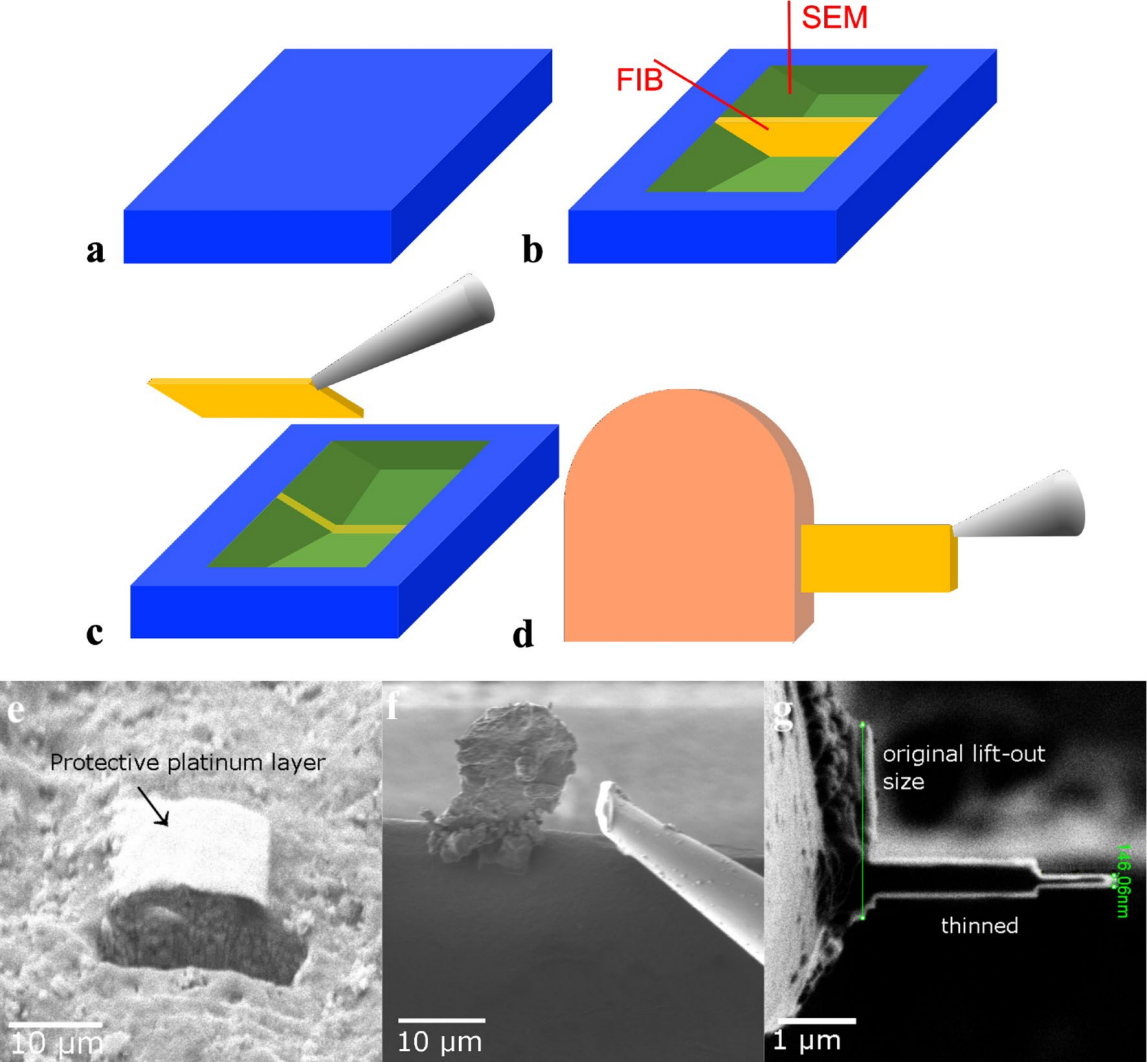
Schematic and example secondary electron images of kerogen lift-out preparation process: (**a**) carbon rich area is identified, (**b**) trenches are milled around the site of interest leaving thin lamella, (**c**) lamella is attached to the Omiprobe nanomanipulator tip using platinum deposition and lamella is cut free by milling sides and bottom, (**d**) lamella is attached to copper grid post with platinum deposition, cut free from the tip and both lamella faces are thinned to electron transparency using gallium ion beam; (**e**) site of interest with platinum deposition on top and a milled trench, (**f**) lamella welded to a copper grid post and Omiprobe nanomanipulator tip already cut free, (**g**) top view of a lamella in the process of thinning.

Electron transparent kerogen specimens were prepared using FEI Quanta200 3D DualBeam FIB-SEM system with Omniprobe Model 100.7 in-situ nanomanipulator. Schematic preparation procedure is shown in Fig. 7. Marcellus and Barnett kerogen samples were obtained by selecting a carbon-rich particle out of a pool of pulverized particles. A particle was welded to the Omiprobe tip by platinum deposition, lifted, positioned next to a copper grid post and welded to the post by platinum deposition, after which the tip was cut away. A protective strip of platinum was deposited on a sample’s upper edge to avoid excessive thinning and particle sides were ablated until about 30 nm thickness lamella was obtained. Utica specimen was extracted by milling trenches around the site of carbon rich area leaving thin lamella, which was then attached to the Omiprobe tip using platinum deposition and cut free from a particle by milling sides and bottom. Utica’s lamella was also attached to the copper grid post and thinned. Gallium ion beam at 30 kV voltage and current between 5 nA (bulk milling) and 0.5 nA–50 pA (for lamella polishing) was used. Cross-sectional samples were examined in JEOL 2100 + transmission electron microscope (equipped with a Gatan Ultrascan 1000 CCD camera and JEOL STEM detectors) operated at 200 kV. It is known that irradiation can damage carbon nanostructures as well as graphitize or induce formation of spherical structures from amorphous phase^38^, uncertainty is even greater in this case as kerogen was never imaged at this scale, thus original structure was previously unknown. Therefore, to reduce damage possibility, the TEM was operated at a minimum voltage (up to 200 kV) required to collect data and images were carefully inspected for changes as the dose was increased.
